# COVID-19 mortality prediction using ensemble learning and grey wolf optimization

**DOI:** 10.7717/peerj-cs.1209

**Published:** 2023-02-24

**Authors:** Lihua Lou, Weidong Xia, Zhen Sun, Shichao Quan, Shaobo Yin, Zhihong Gao, Cai Lin

**Affiliations:** 1 Department of Burn, Wound Repair and Regenerative Medicine Center, The First Affiliated Hospital of Wenzhou Medical University, Wenzhou, China; 2 Department of Big Data in Health Science, The First Affiliated Hospital of Wenzhou Medical University, Wenzhou, China

**Keywords:** Prediction, Mortality, COVID-19, Machine learning, Ensemble learning, Genetic algorithm, Grey wolf optimization, Data science, Artificial intelligence

## Abstract

COVID-19 is now often moderate and self-recovering, but in a significant proportion of individuals, it is severe and deadly. Determining whether individuals are at high risk for serious disease or death is crucial for making appropriate treatment decisions. We propose a computational method to estimate the mortality risk for patients with COVID-19. To develop the model, 4,711 reported cases confirmed as SARS-CoV-2 infections were used for model development. Our computational method was developed using ensemble learning in combination with a genetic algorithm. The best-performing ensemble model achieves an AUCROC (area under the receiver operating characteristic curve) value of 0.7802. The best ensemble model was developed using only 10 features, which means it requires less medical information so that the diagnostic cost may be reduced while the prognostic time may be improved. The results demonstrate the robustness of the used method as well as the efficiency of the combination of machine learning and genetic algorithms in developing the ensemble model.

## Introduction

Since early 2020, approximately 600 million people have been victims of COVID-19, and more than 6.5 million have lost their lives as a result of the epidemic (Ciotti et al., 2020). The escalation in the number of reported cases has put massive pressure on healthcare systems worldwide, especially when the new virus variants continue to become more infectious (Ciotti et al., 2020). While many people have a moderate, self-recovering form of illness, other people suffer from serious and lethal conditions (Altschul et al., 2020). Identifying whether people have a higher probability of developing serious complications or dying is crucial to gaining insight into this disease (Altschul et al., 2020). Zhou et al. (2020) retrospective cohort analysis found a variety of comorbidities (*e.g*., diabetes, hypertension, and coronary artery disease) whose incidence varied substantially between the recovered and perished people. Also, they found that aged patients scored with higher sequential organ failure assessment (SOFA) values and accumulated D-dimers had a substantially higher inpatient mortality rate (Zhou et al., 2020). SOFA evaluates the functioning of several organ systems in the body. A greater SOFA score indicates a greater probability of death (Shahpori et al., 2011). Furthermore, medical signs and symptoms, such as acute renal damage, acute liver injury, the requirement for mechanical ventilation, increased C-reactive protein (CRP), interleukin-6 (IL-6), lymphocyte count, and procalcitonin levels have been identified as additional markers of poor outcome (Yang et al., 2020, Richardson et al., 2020; Cheng et al., 2020; Wu et al., 2020; Altschul et al., 2020). COVID-19 not only causes sepsis and multiple organ dysfunctions but also results in a strong inflammatory response to activate systematic multi-vascular thrombosis (Altschul et al., 2020; Huang et al., 2020; Iba et al., 2020). To identify the mortality risk of patients infected with COVID-19, several statistical scores were suggested (Altschul et al., 2020). Since the SOFA score does not account for the extra thrombotic mitigating factors of severe illness (Vincent et al., 1996), the disseminated intravascular coagulation (DIC) score was suggested for use in guiding anti-coagulation for patients with COVID-19 (Thachil et al., 2020). The DIC score was originally created to assist in the growth evaluation of disseminated intravascular coagulation (Taylor et al., 2001; Sivula, Tallgren & Pettilä, 2005; Thachil et al., 2020). DIC is an uncommon but deadly disorder characterized by irregular blood blockage throughout the blood arteries. Infected or injured people having disrupted normal blood blockage may produce DIC (Colman, Robboy & Minna, 1972).

In recent years, the expansion of computing platforms, powerful technologies, and enormous databases has prompted scientists to devote more time and effort to the development of advanced computational approaches to address multiple problems in a variety of fields. Machine learning and deep learning have become effective analytic tools for investigating the data narratives of numerous domains in order to support decision-making (Deng & Yu, 2014; Jordan & Mitchell, 2015). To estimate the mortality risk score, besides traditional mathematical modeling methods, several computational frameworks were introduced with surprising performance. An enormous number of people infected and killed by COVID-19 has strongly motivated scientists to develop prediction frameworks to assess the mortality risk of COVID-19-infected patients based on their clinical data collected from various periods. To quantitatively evaluate the survival and mortality of matured patients admitted to the intensive care unit (ICU) from at least 48 h to 7 days, Covino et al. (2020) gathered the matured patients’ clinical physiological data during their hospitalized time from March 1 to April 15, 2020. Their findings indicated that the Early Warning Score may accurately predict the risk of severe illness and mortality under the circumstances of high demand for medical evaluation and triage in emergency rooms (Covino et al., 2020). Allenbach et al. (2020) employed a logistic regression model to predict differences in patients’ symptoms during 14-day hospitalized period. Their results pointed out that older age, more impaired respiratory functions, higher CRP levels, and lower lymphocyte counts were related to an elevated risk of ICU admission or high mortality (Allenbach et al., 2020). Estiri et al. (2021) designed a full-staged machine learning framework for predicting hospitalizations, ICU admissions, mechanical ventilation demands, as well as mortality risk based on patients’ historical medical information. Their analysis showed that cardiovascular diseases, a history of neurological illness, and other chronic diseases (*e.g*., diabetes and renal disorders) may contribute to greater mortality (Estiri et al., 2021). Yadaw et al. (2020) analyzed 3,841 cases sourced from Mount Sinai Health System with the reported time of between March 9 and April 6, to construct a mortality prediction model using diverse information including personal information (*e.g*., the patient’s age), testing results (*e.g*., minimum oxygen saturation), and kind of patient contact (inpatient, outpatient, and telehealth visits). Altschul et al. (2020) proposed a scoring metric to predict severity and death of COVID-19-infected case with the expectation of helping doctors prognosticate patients’ condition. Wang et al. (2021) created a prediction model using the same data as Altschul et al.’s study to estimate entry mortality risk of COVID-19-infected cases. Smolderen et al. (2021) investigated all COVID-19 hospital admission cases in the Yale region in 2020 and found that patients with peripheral vascular disease had a greater mortality risk and serious adverse cardiovascular illness than other patients without background diseases. Besides, several meta-analysis studies were conducted to reveal the risk factors leading to higher mortality (Lippi, Lavie & Sanchis-Gomar, 2020; Gerayeli et al., 2021). Information on discovered risk factors is essential to the modeling stage due to their significant contribution to the model’s efficiency.

In this study, we propose an effective computational model to identify the mortality risk of COVID-19-infected patients using ensemble learning in combination with a genetic algorithm (Katoch, Chauhan & Kumar, 2021). The variety of machine learning algorithms is initially surveyed to find the most suitable base classifiers for constructing an ensemble model. The grey wolf optimization (Rezaei, Bozorg-Haddad & Chu, 2018), a member of the family of genetic algorithm, is used to optimize the weight of each base model to boost the performance of the ensemble model. The weighted ensemble strategy was used in several studies (Li et al., 2016; Nguyen et al., 2022). The objective of aggregating several learners *via* an ensemble strategy is to create more a stronger framework by capturing the underlying data distribution more precisely. Our ensemble model is expected to be a simple but effective computational framework that is dependent on small feature sets to come up with robust predictions.

## Materials and Methods

### Dataset

The dataset comes from a study by Altschul et al. (2020). The dataset contains 4,711 COVID-19-infected cases confirmed by a real-time analyzer (characterized by reverse transcriptase-polymerase chain reaction) in the period between March 1 to April 16, 2020. Cases evaluated in the emergency zone but not admitted or died at the site were removed from the study. The majority of patients had a single hospitalization, and the research only evaluated the most recent cases for those with repeated hospitalizations over the study period (Altschul et al., 2020).

**Figure 1:**
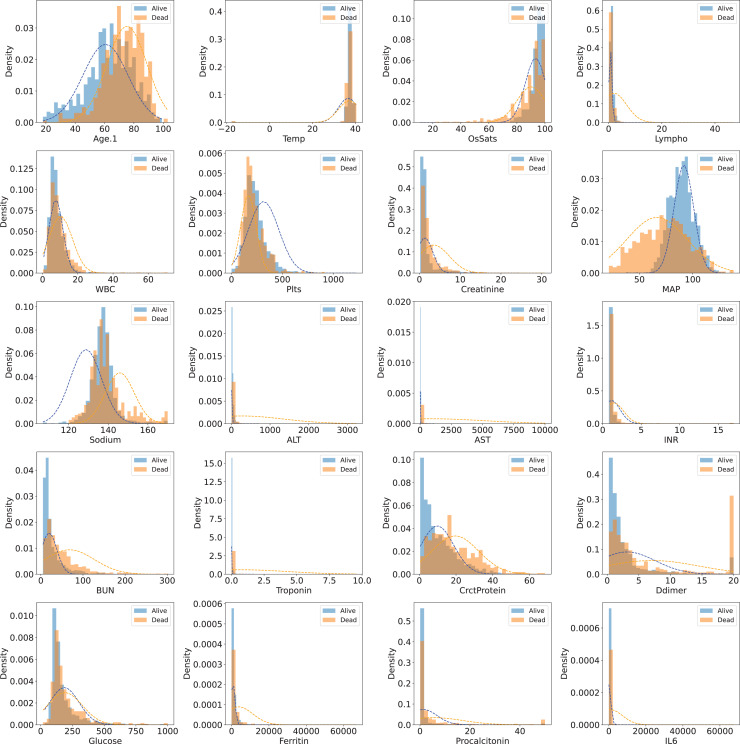
Distribution of 20 selected features in the training set (Cohort 1)

The dataset has 85 variables, most of which are secondary variables created from the original ones. In our study, we used original variables only, so all secondary variables were removed. The “*Derivation cohort*” variable is a categorical variable used for grouping. Samples having “*Derivation cohort* = 1” were used as training samples, while samples having “*Derivation cohort* = 0” were used as validation (25%) samples and test samples (75%). The “*Death*” variable is the target class with two values: 0 (dead) and 1 (alive). Variables whose majority of values were missing were also removed. After filtering the whole set of variables, 20 original variables (features) were obtained for model development. These original variables were “Age.1” (patient’s age), “Temp” (Temperature), “OsSats” (oxygen saturation), “Lympho” (lyphocyte count), “WBC” (white blood cell count), “Plts” (platelet count), “Creatinine” (creatinine level), “MAP” (mean arterial pressure, in mmHg), “Sodium” (sodium level), “ALT” (alanine transaminase level), “AST” (aspartate transaminase level), “INR” (international normalised ratio), “BUN” (blood urea nitrogen), “Troponin” (troponin level), “CrctProtein”, “Ddimer” (D-dimer level), “Glucose” (Blood glucose level), “Ferritin” (ferritin level), “Procalcitonin” (procalcitonin level), “IL6” (interleukin-6 level). Table 1 shows descriptive statistics for 20 selected features (variables) in the two cohorts. Figure 1 visualizes the distributions of those features in the training set (*Cohorts 1*).

**Table 1:** Descriptive statistics for 20 selected features in the data.

Variable	Cohort 0			Cohort 1			Samples with missing data	
	*N*	Mean	SD	*N*	Mean	SD	Count	Ratio
Age.1	2,357	58.6	17.14	2,354	72.55	13.05	0	0.00
Temp	2,281	37.13	3.33	2,277	36.93	5.06	153	0.03
OsSats	2,277	93.69	7.05	2,267	88.71	11.62	167	0.04
Lympho	2,234	1.52	5.97	2,298	1.15	2.54	179	0.04
WBC	2,234	9	8.35	2,298	9.42	5.86	179	0.04
Plts	2,234	242.16	107.3	2,294	218.1	101.75	183	0.04
Creatinine	2,224	1.79	2.53	2,297	2.71	3.02	190	0.04
MAP	2,234	89.02	13.91	2,255	73.24	22.5	222	0.05
Sodium	2,204	137.94	7.23	2,271	139.53	9.16	236	0.05
ALT	2,192	46.95	94.13	2,263	62.68	234.07	256	0.05
AST	2,172	62.49	127.89	2,234	115.97	500.28	305	0.06
INR	2,041	1.18	0.72	2,168	1.36	1.25	502	0.11
BUN	2,034	26.84	28.5	2,072	50.08	42.41	605	0.13
Troponin	1,963	0.04	0.12	2,107	0.15	0.66	641	0.14
CrctProtein	1,867	11.46	10.69	1,968	18.39	11.93	876	0.19
Ddimer	1,761	3.86	5.41	1,849	7.11	7.28	1,101	0.23
Glucose	1,647	175.56	120.03	1,691	201.81	135.22	1,373	0.29
Ferritin	1,608	1,333.1	3,161.78	1,696	2,185.21	4,467.89	1,407	0.30
Procalcitonin	1,462	1.72	6.07	1,535	5.46	11.56	1,714	0.36
IL6	986	235.99	4,008.91	1,089	528.63	4,229.52	2,636	0.56

### Overview of the method

**Figure 2:**
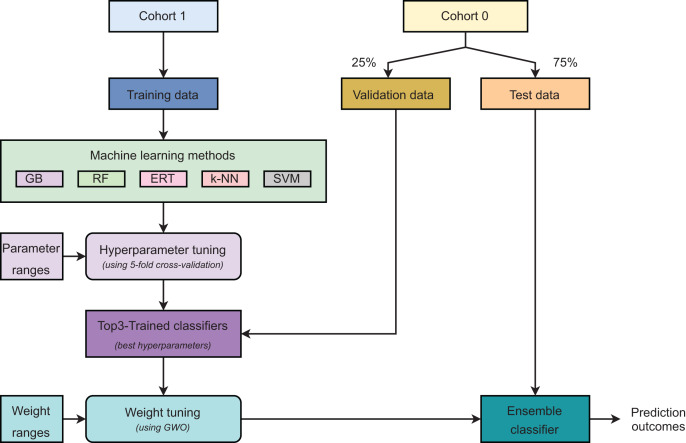
Major steps in model development.

Figure 2 describes the main steps in developing our ensemble model. Initially, samples were grouped into two cohorts: *Cohort 0* and *Cohort 1*. The samples in *Cohort 1* were used as training data. For *Cohort 0*, the validation and test sets were created using random sampling with the ratio of 25% and 75% of total samples, respectively. It is worth noting that the validation set was used to find the optimal weights for selected base classifiers only. A $5$-fold cross-validation was executed on the training set to discover the optimal hyper-parameters for each model. Five learning algorithms, including gradient boosting (GB), random forest (RF), extremely randomized trees (ERT), $k$-nearest neighbors ( $k$-NN), and support vector machines (SVM), were used to construct prediction models and these models were ranked based on their performances. Three best-performing models were selected as the base classifiers for the ensemble learning strategy. The top three classifiers were then retrained on the whole training set using their optimal hyper-parameters. Our proposed strategy is weighted ensemble learning which means each base model is allocated a weight values ( $w_1$, $w_2$, and $w_3$) explored by using the grey wolf optimization (GWO) algorithm (Rezaei, Bozorg-Haddad & Chu, 2018) on the validation set. Eventually, the test set was used to evaluate the model’s performance. The ensemble model predicts a probability for each sample in the form of $p_{ensemble}$ = $w_1$. $p_1$ + $w_2$. $p_2$ + $w_3$. $p_3$.

### Machine learning algorithms

#### Gradient boosting

Gradient boosting (GB) (Friedman, 2001, 2002) is a supervised ensemble learning technique based on the idea that a poor learner may be improved to become better. The “boosting” task involves filtering observations, keeping only those that the weak learner can manage, and then training additional weak learners to handle the remaining challenging data. All “boosting” methods may be generalized to tackle regression, multi-class classification, and diverse problems by allowing the use of arbitrary differentiable loss functions.

#### Random forest

Random forest (RF) (Breiman, 2001), a resilient and efficacious supervised ensemble learning method, was developed based on the “bagging” concept (Breiman, 1996) and random feature selection (Ho, 1995). This approach is characterized by the generation of numerous decision trees that are fairly distinct from one another. The produced outputs are adjusted to subject to either the mode of the classes or the average of the projected values of the multiple trees involved to cope with classification or regression issues, respectively. RF can successfully overcome the primary flaw of the decision tree algorithm known as “overfitting propensity towards the training data.”

#### Extremely randomized trees

Extremely randomized trees (ERT) (Geurts, Ernst & Wehenkel, 2006) is a supervised tree-based ensemble learning method that can address classification and regression issues. The core of this method is a process of significantly randomizing the selection of both the variables and the cut points used to divide a tree node. In the most extreme scenario, it constructs trees whose structures are completely random and unrelated to the output values of the training sample. The parameter allows the randomization intensity to be adjusted to meet the needs of a given situation. The algorithm’s biggest advantage, apart from accuracy, is an improvement in computing speed compared to random forest, another tree-based approach.

#### $ k$-nearest neighbors

$k$-nearest neighbors ( $k$-NN) is one of the most frequently used distance-based supervised learning methods. It was first presented by Fix & Hodges (1989) in 1951 and later developed in its complete form by Altman (1992) in 1992. $k$-NN can be implemented to tackle not only classification but also regression problems. For an unknown sample, class or value can be assigned to it after the algorithm calculates total distances from the its positions to $k$ nearest neighbors. As a distance-based algorithm, it requires data normalization to avoid substantial biases caused by different scales of variables.

#### Support vector machines

Support vector machines (SVM) (Cortes & Vapnik, 1995) is one of the most robust supervised learning algorithms. SVM was first developed to address binary classification tasks before being expanded to cover multi-class classification issues. The SVM’s main idea is to construct a $n$-dimensional hyperplane where $n$ is the number of variables involved. The hyperplane is optimized to maximize the distance between the two farthest data points of two or more classes. Margin distance maximization, in other words, aims to push the data points of each class away from those of the other classes, so that help improving the classification boundary.

#### Hyperparameters tuning

In order to determine the optimal hyperparameters for each model, we conducted $5$-fold cross-validation which is an exhaustive searching process across selected parameter value grids. To fine-tune the GB, RF, ERT, $k$-NN, and SVM-based classifiers, we chose a set of important parameters for each model (Table 2).

**Table 2:** Model parameters and searching range.

Model	Parameter	Searching range
GB	n_estimators learning_rate max_depth min_sample_leaf subsample	[100, 300] {0.01, 0.05, 0.1, 0.2}{2, 3, 4, 5}{1, 2, 3}{0.4, 1.0}
RF	n_estimators max_depth max_features min_sample_leaf	[100, 300] {2, 3, 4, 5} [0.4, 1.0] {1, 2, 3}
ERT	n_estimators max_depth max_features min_sample_leaf	[100, 300] {2, 3, 4, 5} [0.4, 1.0] {1, 2, 3}
$k$-NN	n_neighbors p	[3, 20] {1, 2}
SVM	C gamma	{0.001, 0.005, 0.01, 0.05, 0.1, 1, 10, 100, 1000} {0.001, 0.005, 0.01, 0.05, 0.1, 1, 10, 100, 1000}

### Grey wolf optimization

The grey wolf optimization (GWO) (Rezaei, Bozorg-Haddad & Chu, 2018) technique was inspired by the cooperative hunting behavior of grey wolves. It was first presented by Mirjalili, Mirjalili & Lewis (2014) as an efficient optimization technique among other novel techniques of meta-heuristic optimization family. Later in 2015, Gholizadeh (Gholizadeh, 2015) continued to develop the GWO method to address the nonlinear behavior of a double-layer grid optimization issue. His research revealed that GWO accomplished a more satisfactory job than other algorithms at discovering the most promising strategy for nonlinear double-layer grids.

GWO quantitatively simulates the social order of wolves in their hunting activities with three wolves: *Alpha* ( $\alpha$), *Beta* ( $\beta$), and *Delta* ( $\delta$) whose leadership are decending. These $\alpha$, $\beta$, and $\delta$ wolves represent the best solution, the second-best and third-best solutions, respectively. The remaining wolves in the herd (other potential solutions) are called *Omega* ( $\omega$). In a hunting activity (optimization), $\alpha$, $\beta$, and $\delta$ wolves manage the hunting while $\omega$ wolves follow their leaders. The GWO is characterized by three stages: (i) *Encircling*, (ii) *Hunting*, and (iii) *Attacking*. In stage *Encircling*, the mathematical models for encircling behavior are expressed as:

(1) $${\vec{D} = |\vec{C}.\vec{X}_p(t) - \vec{X}(t)|,}$$

(2) $${\vec{X}(t+1) = \vec{X}_p(t) - \vec{A}.\vec{D},}$$

where $t$ is the present iteration, $\vec {X}$ and $\vec {X}_p$ refer to the location vectors of the grey wolf and the prey, respectively. $\vec {A}$ and $\vec {C}$ are coefficient vectors which are calculated as:

(3) $${\vec{A} = 2.\vec{a}.r_1 - \vec{a},}$$

(4) $${\vec{C} = 2.\vec{r}_2,}$$

where $\vec {r}_1$ and $\vec {r}_2$ are vectors that are randomly initialized in the interval of between 0 and 1, and vector $\vec {a}$ is set to be decreased linearly from 2 to 0. A grey wolf at location (*X*,*Y*) may move to the location of its prey ( $X^*$, $Y^*$) using the preceding equations. In stage *Hunting*, after the grey wolves recognize their prey’s location and encircle them, the $\alpha$ wolf drives the hunting activity. The $\beta$ and $\delta$ wolves sometimes participate in the activity. The hunting behavior of the grey wolves is formulated as:

(5) $${\vec{D_\alpha} = |\vec{C_1}.\vec{X}_\alpha - \vec{X}|,}$$

(6) $${\vec{D_\beta} = |\vec{C_2}.\vec{X}_\beta - \vec{X}|,}$$

(7) $${\vec{D_\delta} = |\vec{C_3}.\vec{X}_\delta - \vec{X}|,}$$

(8) $${\vec{X_1} = \vec{X_\alpha} - \vec{A_1}.\vec{D}_\alpha,}$$

(9) $${\vec{X_2} = \vec{X_\beta} - \vec{A_2}.\vec{D}_\beta,}$$

(10) $${\vec{X_3} = \vec{X_\delta} - \vec{A_3}.\vec{D}_\delta,}$$

(11) $${\vec{X}(t+1) = \frac{\vec{X_1} + \vec{X_2} + \vec{X_3}}{3}.}$$

In an $n$-dimensional investigation space, the search agent uses these equations to adjust its location based on $\alpha$, $\beta$, and $\delta$. The investigation space parameters $\alpha$, $\beta$, and $\delta$ would constitute a circle, and the ultimate location is estimated inside that circle. By the other way of explanation, $\alpha$, $\beta$, and $\delta$ wolves estimate the prey’s location, whereas other wolves in the herd update their locations to approach their prey. In stage *Attacking*, after the prey (objective) stops moving, the grey wolves finish their hunting game by attacking the prey.

### Ensemble learning strategy

To construct the ensemble model, the three best-performing classifiers (from $5$-fold cross-validation) were selected and termed as ‘*base classifiers*’. For each data point, the ensemble model’s predicted probability is derived by successively multiplying the probabilities predicted by the three base classifiers by their respective weights.

(12) $${p_{ensemble, i} = w_1.p_{1, i} + w_2.p_{2, i} + w_3.p_{3, i},}$$

where $p_{ensemble, i}$ is the obtained ensemble model’s probability; $w_1$, $w_2$, and $w_3$ are the weights of the three base classifiers; $p_{1, i}$, $p_{2, i}$, and $p_{3, i}$ are the predicted probabilities computed by the three base classifiers. The sum of the weights is equal to 1 and these weights are computed by the GWO algorithm to maximize the area under the receiver operating characteristic (ROC) curve (AUC) value on the validation set.

## Results and discussions

The model’s performance was evaluated using multiple metrics: balanced accuracy (BA), sensitivity (SN), specificity (SP), precision (PR), Matthews’s correlation coefficient (MCC), and the area under the receiver operating characteristic curve (AUC).

### Five-fold cross-validation

To discover the optimal hyperparameters for individual model, we used a $5$-fold cross-validated randomized search across parameter settings. During the $5$-fold cross-validation, the first four folds were employed as the training fold, while the left fold was employed as the validation fold. Each fold was iteratively used as a test fold until no unused fold was left. Table 3 provides results of the hyperparameter tuning using $5$-fold cross-validation. The results indicate that the GB model achieves the highest cross-validated AUC value, followed by the RF and ERT classifiers. The $k$-NN and SVM classifiers have similar cross-validated performance. Hence, the GB, RF, and ERT classifiers were selected as base classifiers for the ensemble learning strategy. The GB, RF, and ERT classifiers were finally retrained on the training set using their tuned hyperparameters.

**Table 3:** $5$-fold cross-validation on the training set.

Model	AUC	Tuned hyperparameters
GB	0.9054	n_estimators = 290 learning_rate = 0.05 max_depth = 3 min_sample_leaf = 2 subsample = 0.66
RF	0.8798	n_estimators = 108 max_depth = 4 max_features = 0.75 min_sample_leaf = 2
ERT	0.8770	n_estimators = 294 max_depth = 4 max_features = 0.84 min_sample_leaf = 1
k-NN	0.8632	n_neighbors = 19 p = 1
SVM	0.8632	C = 1 gamma = 0.1

### Ensemble model

The trained GB, RF, and ERT models were termed ‘*model’* numbered *1*, *2*, and *3*, respectively. The validation set was used to optimize the model’s weights ( $w_1$, $w_2$, and $w_3$) that contributed to the ensemble model’s prediction. The ensemble model’s prediction has the form of $p_{ensemble}$ = $w_1$. $p_1$ + $w_2$. $p_2$ + $w_3$. $p_3$ where $p_1$, $p_2$, and $p_3$ are the predicted probabilities of ‘*model*’ *1*, *2*, and *3* respectively. The ensemble model was created with three scenarios using the all-features set and two selected-features sets. An ensemble model constructed from a smaller feature set while still giving a similar performance is preferable. Table 4 gives information on base classifiers and their tuned weights in three ensemble learning scenarios.

**Table 4:** Base models and their tuned weights in three ensemble learning scenarios.

Ensemble scenario	Number of features	Model	Tuned weight
All features	20	GB	0.5950
		RF	0.0687
		ERT	0.3363
10 selected features	10	GB	0.6900
		RF	0.0792
		ERT	0.2308
5 selected features	5	GB	0.4198
		RF	0.0893
		ERT	0.4909

#### Ensemble model with all features

**Figure 3:**
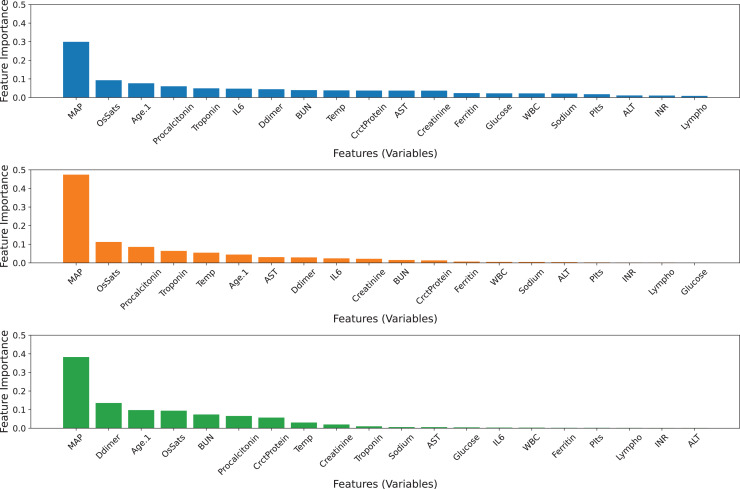
Feature importance ranking of the three base classifiers across 20 features. (A) GB model, (B) RF model, (C) ERT model.

All 20 features were used in the construction of three base classifiers and an ensemble model. Table 5 displays the performance of the three base classifiers and the ensemble model. The ensemble model obtains AUC values of 0.7801 and MCC values of 0.3898, which are greater than those of its base classifiers. The GB model achieves higher balanced precision and sensitivity than the others. The ERT model outperforms other classifiers in terms of accuracy and specificity. Based on the tuned values, the GB model contributes the most to the ensemble model’s predictive ability, followed by the ERT and RF classifiers (Table 4). Figure 3 visualizes the feature importance ranking of three basic classifiers across 20 features. Variable ‘*MAP*’ has the greatest effect on all classifiers. Moreover, the ‘*OsSats*’ variable is ranked among the top five key features in three classifiers. While the ‘*Age.1*’ variable is regarded as the third most significant feature by the GB and ERT classifiers, it has less of an effect on the RF classifiers. The GB and RF classifiers rank ‘*Troponin*’ among their top five key features, but the ERT model mostly disregards it. In addition, there are variables whose contributions may be ignored by the RF and ERT classifiers.

**Table 5:** The performance on the test set of the models developed using 20 selected features.

Model	AUC	BA	SN	SP	PR	MCC
GB	0.7768	0.6828	0.4734	0.8921	0.5582	0.3885
RF	0.7598	0.6377	0.3519	0.9235	0.5697	0.3325
ERT	0.7695	0.6156	0.2785	0.9526	0.6286	0.3223
Ensemble	0.7801	0.6726	0.4304	0.9147	0.5923	0.3898

#### Ensemble model with 10 selected features

**Figure 4:**
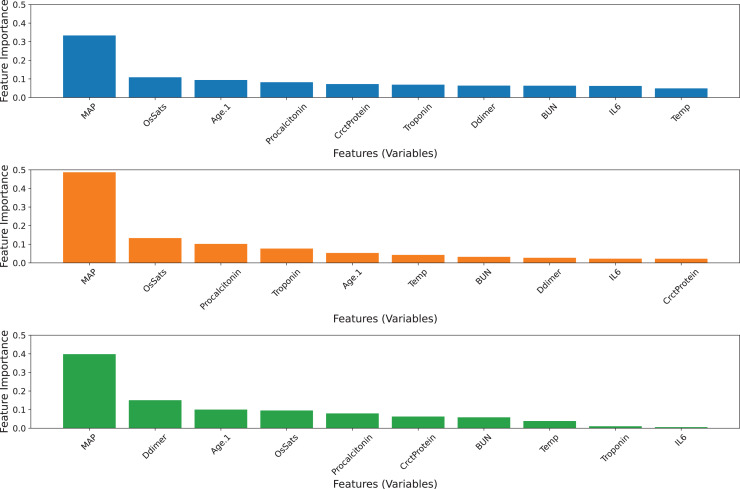
Feature importance ranking of three base classifiers across 10 features. (A) GB model, (B) RF model, (C) ERT model.

The ensemble model was developed by selecting the best five features of each base model (Fig. 3). Since the top-five features of the three base classifiers are somewhat different from each other, all features in the top five were preserved. Hence, 10 features were used to build three basic classifiers and an ensemble model. In terms of the AUC value, the ensemble model remains superior to its individual classifiers. The GB model exhibits a superior balanced accuracy of 0.6844, a sensitivity of 0.4759, and an MCC of 0.3920 (Table 6). Comparatively, the ERT model achieves greater specificity and accuracy compared to the other classifiers. Based on the tuned values, the GB model still contributes the most to the ensemble model’s predictive ability, followed by the ERT and RF classifiers (Table 4). However, the contribution of the ERT model decreases while the contribution of RF increases. Figure 4 visualizes the feature importance ranking of three basic classifiers across 10 selected features. The top-three features of the three base classifiers in the first and second scenarios are unchanged. The performance of ensemble models developed with 10 and 20 features has equivalent predictive efficiency.

**Table 6:** The performance on the test set of the models developed using 10 selected features.

Model	AUC	BA	SN	SP	PR	MCC
GB	0.7783	0.6844	0.4759	0.8929	0.5612	0.3920
RF	0.7626	0.6489	0.3772	0.9206	0.5775	0.3513
ERT	0.7694	0.6135	0.2810	0.9461	0.6000	0.3090
Ensemble	0.7802	0.6725	0.4405	0.9045	0.5705	0.3804

#### Ensemble model with five selected features

The ensemble model was created by picking the top three features of each base model (Fig. 4). Since the top five features of the three base classifiers are distinct, the top five features were all retained. The performance of the ensemble model and GB model drops while those of RF and ERT classifiers increase. Although the ensemble model still has the greatest AUC value compared to other classifiers (Table 7). The contribution of the GB model significantly decreases. While the contribution of the ERT model double and that of the RF model slightly increases. Since the performance of the ensemble model is closely associated with the performance of the GB model, the decline in the performance of both predictors is explainable.

**Table 7:** The performance on the test set of the models developed using five selected features.

Model	AUC	BA	SN	SP	PR	MCC
GB	0.7734	0.6647	0.4430	0.8863	0.5287	0.3517
RF	0.7657	0.6496	0.3772	0.9220	0.582	0.3542
ERT	0.7728	0.6056	0.2608	0.9504	0.6023	0.2976
Ensemble	0.7782	0.6523	0.3899	0.9147	0.5683	0.3522

#### Model’s robustness and stability

**Figure 5:**
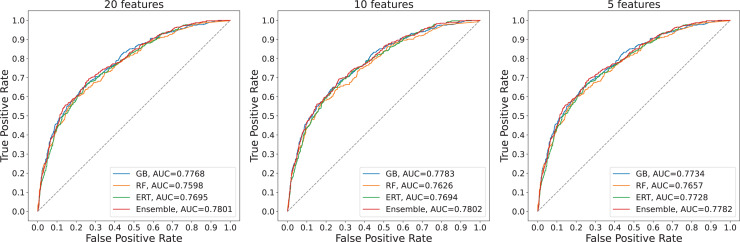
ROC curves of the models corresponding to different numbers of features used.

To assess the model’s robustness and stability, we repeated the experiments 10 times for each model. Table 8 provides information on the model performance of three models over ten random trials. Results show that the performances of the three models have small variations and high repeatability. The performances of the model developed with 10 features and the model developed with 20 features are equivalent while the performance of the model developed with five features is smaller. The repeated experimental outcomes confirm the model’s robustness and stability. Besides, the model developed with 10 features is the best-performing model with a small number of variables used but still has high predictive power. Figure 5 visualizes the ROC curves of base classifiers and ensemble model.

**Table 8:** The performance on the test set of three models over ten random trials.

Trial	AUCs of models using		
	20 Features	10 Features	5 Features
1	0.7801	0.7802	0.7782
2	0.7800	0.7799	0.7766
3	0.7800	0.7798	0.7766
4	0.7799	0.7798	0.7766
5	0.7800	0.7798	0.7766
6	0.7800	0.7799	0.7766
7	0.7800	0.7798	0.7766
8	0.7799	0.7800	0.7766
9	0.7800	0.7798	0.7767
10	0.7799	0.7798	0.7766
Mean	0.7800	0.7800	0.7768
SD	0.0001	0.0001	0.0005
95% CI	[0.7799–0.7800]	[0.7798–0.7800]	[0.7764–0.7771]

## Conclusions

According to the outcomes of three ensemble learning strategies, the ensemble learning model developed with 10 features (the top five features of each base model) is the optimal approach for constructing the model to identify the mortality risk of COVID-19 patients. The model in the first scenario (using 20 features) has the same performance as the model in the second scenario (using 10 features), but the model in the second scenario utilizes fewer features, which means less information is needed in the risk prognosis. The reduction of medical information used in predicting mortality risk can improve prognostics as well as save a large budget on laboratory testing. The grey wolf optimization is a fast, robust, and effective optimization technique to explore the suitable contributing weights for each base model to promote the prediction power in the ensemble learning strategy. Repeated experiments confirm the model’s robustness and stability. However, the proposed method has drawbacks, such as the inability of finding globally optimized weight and the non-controlling of stochastic-based process, that need to be addressed in the future.

## Supplemental Information

10.7717/peerj-cs.1209/supp-1 Supplemental Information 1 Code and data used in experiments.

## Additional Information and Declarations

### Data Availability

The following information was supplied regarding data availability: The data used in this study is available at Figshare: Lin, Cai (2022): covid_19.xlsx. figshare. Dataset. https://doi.org/10.6084/m9.figshare.21739745.v1. The raw data is available in the Supplemental Files. The code is available at Zenodo: zhaoyipchang. (2022). zhaoyipchang/covid19_mortality_gwo: 1.0 (1.0). Zenodo. https://doi.org/10.5281/zenodo.7472400.
